# Veratramine influences the proliferation of human osteosarcoma cells through modulating the PI3K/AKT signaling cascade

**DOI:** 10.1016/j.gendis.2025.101630

**Published:** 2025-04-11

**Authors:** Zhou Xie, Xiao Qu, Ziyun Li, Yingtao Duan, Yafei Zhu, Jiayu Wang, Xueqian Han, Jun Zhang, Jinyong Luo, Xiaoji Luo

**Affiliations:** a Department of Orthopedics, The First Affiliated Hospital of Chongqing Medical University, Chongqing 400016, China; b Department of Orthopedics, The First Affiliated Hospital of Chongqing Medical and Pharmaceutical College, Chongqing 400060, China; c Key Laboratory of Diagnostic Medicine Designated by the Chinese Ministry of Education, Chongqing Medical University, Chongqing 400016, China

**Keywords:** Anti-tumor, Network pharmacology, Osteosarcoma, PI3K/AKT, Veratramine

## Abstract

Osteosarcoma (OS) is a highly aggressive tumor with a propensity for early metastasis. Current treatment methods, such as chemotherapy, often bring significant side effects, affecting patients' quality of life. Veratramine (VER), an alkaloid derived from the American lily plant, has shown potential in cancer treatment. This study looks at the effects and mechanisms of VER on osteosarcoma. VER's impact was assessed using a variety of procedures, including crystal violet staining, the CCK-8 assay, and the colony formation assay, which measured cell proliferation. Wound healing assay and transwell assay were employed to evaluate the migration and invasion of osteosarcoma cells. Hoechst33258 staining, flow cytometry, and transmission electron microscopy were used to investigate apoptosis. Protein expression was assessed using western blotting and immunofluorescence. Blood tests and hematoxylin-eosin staining were used to establish VER's *in vivo* safety, and its effectiveness was proven using an orthotopic tumor model. The results showed that VER greatly decreased osteosarcoma cell growth, migration, and invasion while inducing apoptosis. Animal tests confirmed these findings, confirming VER's high efficacy and safety *in vivo*. VER might function by inhibiting the PI3K/AKT signaling pathway. To sum up, VER shows promise in treating osteosarcoma by exhibiting significant anti-tumor activity in laboratory and animal studies, likely through the regulation of the PI3K/AKT signaling pathway.

## Introduction

Osteosarcoma (OS) is a dangerous bone tumor that primarily affects the femur, tibia, and humerus in youngsters. With an annual occurrence of 2–3 cases per million, this condition is extremely aggressive and likely to spread, resulting in swift disease advancement, unfavorable outcomes, and a high death rate.^1^^,^^2^ Currently, neoadjuvant chemotherapy, radical surgery, and chemotherapy after surgery are included in standard treatment protocols.^3^ Despite the substantial improvement in survival rates due to neoadjuvant chemotherapy, only 25%–30% of patients with metastatic or recurrent OS survive long-term.^4^ There has been a paucity of therapy choices over the last 40 years, and regularly used chemotherapeutic medications like cisplatin, methotrexate, and doxorubicin, while effective, nevertheless represent a risk of nephrotoxicity, ototoxicity, and neurotoxicity.^5^ The study of personalized treatment approaches, including targeted therapy, immunotherapy, and antibody–drug conjugates over the past few years has shown promising results, but they remain in the early phases of development.^6^ It is therefore imperative that new, safe, and effective drugs are developed to improve OS treatment and prognosis.

Because of their low toxicity and great efficacy, natural active compounds extracted from plants are becoming a key source of anti-tumor chemotherapeutic medications.^7^^,^^8^ There is a naturally occurring alkaloid compound called veratramine (VER) that is extracted from plants of the veratrum plant genus in the lily plant family.^9^ This substance exhibits significant biological activities, including anti-tumor, anti-inflammatory, anti-hypertensive, anti-thrombotic, and neuroprotective properties.^10^ VER has been linked to anti-tumor effects in numerous studies. Kim et al found that VER inhibited the MDM2/p53/p21 pathway, which regulates glioma cell development.^11^ Park et al discovered that VER decreased cell cycle progression, migration, and invasion in androgen-independent prostate cancer cells.^12^ HepG2 cells treated with VER also exhibit autophagic cell death.^13^ Additionally, VER inhibits the downstream signaling route of the transcription factor activator protein-1 (AP-1), which controls several cellular functions such as cell growth, programmed cell death, and epithelial–mesenchymal transition (EMT).^14^ VER may be an effective and safe anti-tumor drug, according to these studies. While studies have shown VER to be anti-cancer in liver cancer, prostate cancer, and glioma, the effect of VER on OS progression remains unclear.

The onset and progression of OS are tightly related to a number of intracellular signaling pathways.^15^ Increased phosphoinositide 3-kinase (PI3K) signaling is thought to be a characteristic of cancer. Research has revealed that by modifying the activation status of downstream effector molecules, this signaling pathway can influence angiogenesis, autophagy, apoptosis, transformation, and cell proliferation.^16^^,^^17^ The PI3K/protein kinase B (AKT) pathway exhibits significant activation in OS cells.^17^^,^^18^ Thus, devising effective therapies for OS necessitates comprehending the control mechanisms of the PI3K/Akt/mechanistic target of rapamycin (mTOR) signaling pathway. Many studies document the anti-OS effects of targeting the PI3K/AKT signaling pathway. One unique strategy for treating OS is using PI3K/AKT signal inhibitors to limit the activation of its effector molecules and increase apoptosis of OS cells.^19^, ^20^, ^21^ This suggests that a new and possibly effective targeted treatment involves disrupting the PI3K/AKT signaling pathway.

This research explored and confirmed VER's safety in living organisms, its ability to suppress OS cells *in vitro* and *in vivo* and its potential molecular mechanisms through network pharmacology. This research aims to offer both experimental and theoretical support for utilizing VER in the treatment of OS.

## Materials and methods

### Cell lines, cell culture, and chemical preparations

143B and HOS human OS cell lines were purchased from the American Type Culture Collection (ATCC). Professor Tongchuan He of the University of Chicago's Department of Molecular Oncology kindly contributed the 143B-Fluc cell line. 143B and 143B-Fluc cells were grown at 37 °C in a humidified environment with 5% CO_2_, using Dulbecco's modified Eagle's medium (DMEM, HyClone, USA) enriched with 10% fetal bovine serum (Excell Bio, China). Minimal essential medium (MEM) was used to cultivate HOS cells. The supplier of VER (purity > 98%) was Chengdu Ruifensi Co., Ltd., China. It was kept at −80 °C after being dissolved in DMSO. VER was dissolved in carboxymethyl cellulose sodium and kept at −20 °C for use in animal research. After being bought from TargetMol in Shanghai, China, the PI3K activator 740-YP was dissolved in DMSO and kept at −80 °C. Specific primary antibodies, including p-AKT from Abclonal (ABclonal Technology Co., Ltd., Wuhan, China), proliferating cell nuclear antigen (PCNA), Bax, Bad, Bcl-2, Snail, vimentin, N-Cadherin, E-Cadherin, and matrix metallopeptidase 2 (MMP-2), were used. The antibody has a 1:1000 dilution.

### Crystal violet assay

24-well plates were planted with 143B and HOS cells. When the cells achieved 30% confluence, they were exposed to various concentrations of VER (0 μM, 30 μM, 40 μM, and 50 μM). We indicate these concentrations with DMSO, L, M, and H in the finding section and figure presentations. Crystal violet staining was applied to the cells after they had been treated for 24 h and 48 h. To fully dissolve the crystal violet for measurement, 10% glacial acetic acid was poured into each well and agitated consistently for 10 min. The absorbance was then measured at 570 nm using a microplate reader.

### CCK-8 assay

143B and HOS cells were planted in 96-well plates at 3000 cells per well and incubated for 24 h. The concentrations of the therapies were administered in accordance with the methods described above. Following 24 h and 48 h of treatment, 10 μL of CCK-8 reagent (TargetMol, China) was added to each well and incubated for 2 h. Subsequently, a versatile microplate reader was employed to measure the absorbance of each sample at a wavelength of 450 nm.

### Colony formation assay

One thousand cells per well of 6-well plates were planted with 143B and HOS cells. Following the formation of 3–4 cell clones, the cells were treated for 7 days with varying amounts of control media and VER (0, 3, 4, 5 μM). Following fixation, crystal violet was used to stain the cells. Finally, Image J was utilized to determine the number of colonies generated.

### Flow cytometry analysis

Six-well plates were planted with 143B and HOS cells. After the cells' confluence reached 30%, they were exposed to various VER concentrations for a whole day. To investigate apoptosis, the cells underwent three rinses with phosphate buffer saline solution (PBS) before being analyzed with flow cytometry and an Annexin V-FITC/PI apoptosis detection kit. To assess the cell cycle, cells were fixed overnight at 4 °C in 500 μL of chilled 75% ethanol before being stained with propidium iodide. Flow cytometry was utilized to evaluate the cell cycle distribution.

### Hoechst33258 staining

24-well plates were planted with 143B and HOS cells. After the cells' confluence reached 30%, they were exposed to various VER concentrations for a whole day. The cells were preserved in 4% paraformaldehyde and then stained using Hoechst 33258. Apoptotic cells were observed and caught at random with an inverted fluorescence microscope.

### Wound healing assays

To create a monolayer, 143B and HOS cells were planted into 6-well plates. After the cells were nearly fully confluent, a clean 10 μL pipette tip was employed to create a synthetic linear wound in the monolayer. Following a PBS rinse, the cells received VER treatment of different concentrations. Using an inverted microscope, the process of wound healing was then captured on camera at 0, 12, and 24 h after the initial damage. Following a random image of each wound, the width was assessed using ImageJ software.

### Transwell assay

To test cell invasion, 60 μL of Matrigel was diluted 20 times and applied to the top chamber. 500 μL of growth medium with various VER concentrations was placed in the lower chamber. 143B and HOS cells (2.5 × 10^4^ cells in 400 μL of serum-free DMEM for 143B and MEM for HOS) were seeded in the upper chamber and cultured for 24 h. After removing non-invading cells from the membrane's upper layer using a cotton swab, cells that had infiltrated the underside were fixed using paraformaldehyde for 15 min, coated with crystal violet for 15 min, and pictured with an optical microscope. The migration assay used a comparable method, except that the upper compartment lacked a Matrigel coating.

### Western blot

After being seeded onto 10 cm culture dishes, 143B and HOS cells were exposed to the different doses of VER for a full day. Cell lysis solution with 1% protease and phosphatase inhibitors (Roche, Switzerland) was used to lyse the cells after trypsinization. The BCA method from Beyotime (Institute of Biotechnology, China) was used to measure the concentration of proteins. Protein samples were separated by SDS-PAGE after being placed onto 10% SDS gels. Following electrophoresis, proteins were moved onto PVDF membranes. Following an overnight incubation at 4 °C with specific primary antibodies, the membranes were then blocked using 5% non-fat milk. Following washing with 1 × TBST three times, the membranes were exposed to horseradish peroxidase-linked secondary antibodies (ZSGB-BIO, China) for 1 h at ambient temperature. Using the ChemiDoc MP Imaging System (BioRad, USA), protein bands were seen and quantified.

### Network pharmacology analysis of veratramine’s effects on osteosarcoma

The 2D structure of VER was sourced from the PubChem database (https://pubchem.ncbi.nlm.nih.gov/). PharmMapper (https://www.lilab-ecust.cn/pharmmapper/), SwissTargetPrediction (https://www.swisstargetprediction.ch/), and the UniProt database (https://www.uniprot.org/) were utilized to identify targets associated with drugs. OS-related targets were sourced from GeneCards (https://www.genecards.org/), TTD (https://db.idrblab.net/ttd/), and OMIM (https://omim.org/). A network of protein–protein interactions was constructed utilizing the STRING database (https://string-db.org/). The “disease-target-component” network diagram was created using Cytoscape 3.9.1, and its MCODE plugin was utilized to cluster the core protein–protein interaction network and create functional modules. To shed further light on the possible workings of VER in the treatment of OS, the CytoNCA plugin was utilized to identify important targets using several techniques. To explore potential biological processes and signaling pathways involved in treating OS with VER, the clusterProfiler package in R software (version 4.0.2) on a Windows platform was utilized to perform the Kyoto Encyclopedia of Genes and Genomes (KEGG) and Gene Ontology (GO) analyses. Keywords for enrichment were chosen based on an adjusted *p*-value threshold below 0.05.

### Molecular docking

The 2D structure file for VER was obtained from the PubChem database (https://pubchem.ncbi.nlm.nih.gov), while the 3D core structures of protein receptors were sourced from the Protein Data Bank (PDB) (https://www.rcsb.org/). The target's PDB IDs are listed in the supplemental table. OpenBabel 2.4.1 and PyMOL 2.2.0 tools were utilized to prepare the 2D structure of VER for processes such as solvation, desolvation, and charge computations, and prepare the target proteins. Using PyRX software, the processed VER with the targets was docked and then the Discovery Studio (DS) software was used to see the proteins.

### *In vivo* toxicity assessment

The toxicity of VER *in vivo* was examined using immunocompetent BALB/c mice. Chongqing Medical University's Institutional Animal Care and Use Committee (IACUC) granted authorization for the animal study. The permission number from the ethics committee is 2024-04087. We summarized existing studies on the *in vivo* use of VER (see Table S1 for listing the concentrations, dosages, and administration routes for related disease models). In this study, we used oral gavage as the administration route.^12^^,^^13^^,^^22^ Due to the first-pass elimination effect, orally administered drugs typically have a bioavailability of only 25%–30% compared with intravenous injection. Therefore, we chose doses of 20 mg/kg, 30 mg/kg, and 40 mg/kg, based on VER's IC50 and its application in other tumors, and administered the drug every other day to observe its inhibitory effects on OS. Six-week-old female BALB/c mice were separated into five groups, each containing three mice, and administered different doses of VER (20 mg/kg, 30 mg/kg, 40 mg/kg) or 0.5% sodium carboxymethyl cellulose via oral gavage every other day for 21 days. Blood was drawn from the orbital cavity and analyzed using an automated hematology device (Sysmex, XT-2000i). The serum was isolated, and the concentrations of aspartate aminotransferase (AST), alanine aminotransferase (ALT), and blood urea nitrogen (BUN) were measured with an automated biochemical analyzer from IDEXX Laboratories, Inc., Westbrook, ME, USA. Following a cervical dislocation, all mice were sacrificed. The tissues from the heart, lungs, spleen, liver, and kidneys of each mouse group were stained to examine their histological structures.

### Veratramine's anti-tumor growth effects *in vivo*

Six-week-old female BALB/c nude mice were sourced from Changzhou Cavens Experimental Animal Co., Ltd. and kept in a pathogen-free environment at the Animal Center of Chongqing Medical University. The Ethics Committee of Chongqing Medical University authorized the animal study (approval number shown above). The nude mice's abdomens and knee joints were cleaned with iodine before anesthesia. An intraperitoneal injection of 2% pentobarbital sodium at a dose of 80 mg/kg was used to induce anesthesia. Each nude mouse received an anesthetic amount ranging from 0.02 to 0.04 mL. A total of 2 × 10^7^ 143B-fluc cells, suspended in 100 μL PBS, were administered into the tibial plateau of the nude mice. Following the injection, the nude mice were randomly divided into five groups, each containing three mice. VER (20, 30, 40 mg/kg) mixed in carboxymethyl cellulose solution was orally administered every two days. The nude mice's body weight and tumor size were tracked throughout the experiment. On day 21, live imagery was used. Mice were given gas anesthesia (*e.g.*, 2% isoflurane) before imaging. An intraperitoneal injection of potassium luciferin (150 mg/kg) was given and let to circulate for 10 min. An *in vivo* imaging system was employed, utilizing suitable excitation and emission filters. Anesthetized animals were put on the imaging bed, and fluorescence imaging data was recorded. IndiGo 2.0.5.0 was used to analyze the data, including quantifying the intensity and spread of the fluorescence. Following imaging, the animals were euthanized with 2% pentobarbital sodium and a significant amount of carbon dioxide. Samples from tumors, lungs, heart, liver, and kidneys were gathered for further analysis.

### Hematoxylin and eosin staining assay

The tumor, heart, lungs, spleen, liver, and kidneys were fixed with paraffin, sectioned into 5 μm slices, and stained with hematoxylin and eosin solution according to the standard protocols. Hematoxylin-eosin stained slices were examined using an optical microscope and photographed at random.

### Immunohistochemistry

The tumor tissue slices were deparaffinized with xylene before being dried with 100%, 90%, and 75% ethanol solutions, washed with PBS, and incubated with primary antibodies at 4 °C overnight. The samples were treated with horseradish peroxidase-linked secondary antibodies at ambient temperature for 30 min after rinsing with PBS. In the end, the protein of interest was detected with a diaminobenzidine substrate kit, and images were captured through a light microscope.

### Immunofluorescence

143B cells were seeded on cell slides and cultivated until they reached 30% confluence. VER was administered at a dosage of 40 μM to the cells. The control group remained untreated. When the cells had reached 70%–80% confluence, they were fixed with 4% paraformaldehyde for 10 min and then washed three times with PBS. The cells were permeabilized for 10 min using 0.2% Triton X-100 and then rinsed three times with PBS. Following a 1-h block with 5% BSA, the primary antibodies were left to incubate at 4 °C overnight. The next day, fluorescently tagged goat anti-rabbit IgG (CY3) secondary antibodies (Servicebio, China) were incubated at room temperature in the dark for 1 h, followed by three PBS washes. DAPI staining was performed for 5 min, followed by three PBS washes. Finally, a drop of Anti-Fade Mounting Medium (Beyotime, China) was applied before covering it with a coverslip. Images were obtained with a confocal microscope (Leica, Germany).

### Transmission electron microscopy

143B cells were exposed to 40 μM of VER for a duration of 24 h. The control group was untreated. The cells were preserved in a 2.5% glutaraldehyde mixture at 4 °C for 2 h. Following fixation, the cells underwent three washes with 0.1 M phosphate buffer, each lasting 15 min. Dehydration was then performed using a succession of ethanol concentrations (50%, 70%, 90%, 95%, and 100%), each lasting 15 min. After drying out, the cells were embedded in acrylic resin and solidified at 60 °C for 48 h. Following embedding, the cells were cut into ultra-thin slices of 70-to-90 nm thickness by an ultramicrotome. The samples were subsequently stained first with 2% uranyl acetate and then with lead citrate, each for 10 min. Ultimately, the specimens were analyzed and imaged using transmission electron microscopy to identify the ultrastructural features of the cells and mitochondria.

### Statistical analysis

Each experiment in this research was performed three times. The information is presented as mean ± standard deviation and analyzed using GraphPad Prism 9. Group differences were assessed with a one-way ANOVA, and Tukey's post hoc test was subsequently used for comparisons between groups. A *p*-value below 0.05 was deemed to be statistically significant.

## Results

### Veratramine reduces the growth of human OS cells

**Figure 1:**
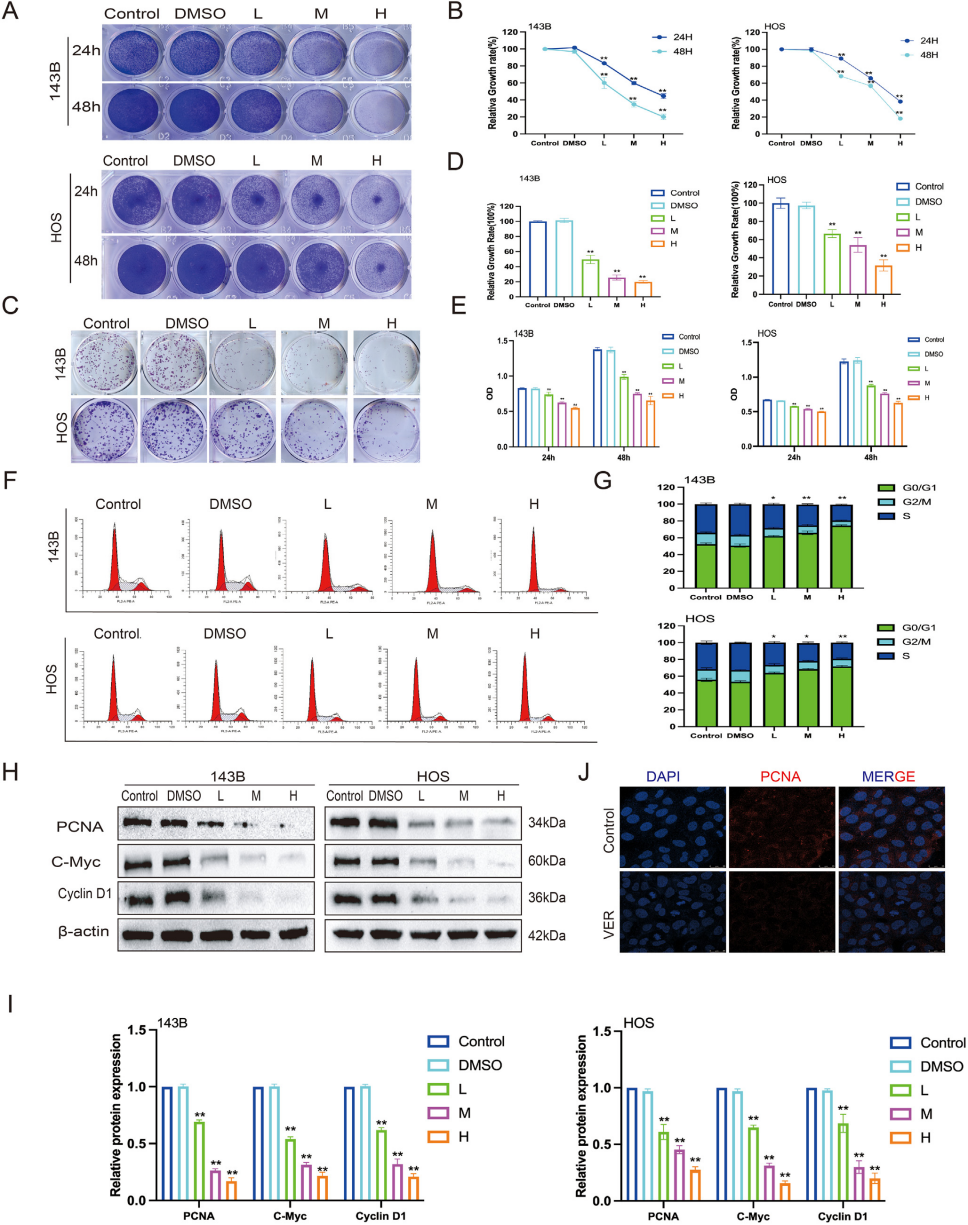
Veratramine inhibits the proliferation of human osteosarcoma cells. **(A**–**E)** The effects of VER on the proliferation of 143B and HOS were detected by the crystal violet staining (A, B), colony formation assay (C, D), and CCK8 assay (E). **(F, G)** The effects of VER on the cell cycle of 143B and HOS were detected by flow cytometry assay. **(H, I)** The protein levels of proliferation-related molecules in 143B and HOS were tested by Western blot. **(J)** PCNA in the cytoplasm of 143B was detected by immunofluorescence test, with PCNA labeled with Cy-3 in red and the nucleus labeled in blue with DAPI. Results are presented as mean ± standard deviation. Statistical significance is indicated by ∗*p* < 0.05 and ∗∗*p* < 0.01 versus the control group.

Firstly, we used crystal violet staining to determine the effects of various VER concentrations on the proliferation of human OS cells 143B and HOS. The results demonstrated that VER significantly inhibited cell proliferation over time and with varying concentrations, in contrast to the control group (Fig. 1A, B). The experiment on colony formation validated that VER suppressed the proliferation compacity of OS cells (Fig. 1C, D). The viability of the human OS cells was then assessed using the CCK8 test. The findings indicated that VER significantly decreased cell survival in a manner dependent on both dosage and time, relative to the control group (Fig. 1E). The IC50 values for 143B and HOS cells at 24 h were 40.90 μM and 46.50 μM, respectively, whereas, at 48 h, they were 34.27 μM and 40.44 μM, respectively.

Additionally, through flow cytometry, we observed that higher drug concentrations led to a greater proportion of cells being arrested in the G0-G1 phase, while the percentage of cells in the S phase consistently decreased compared with the control group. This indicates that VER could suppress OS cells in the G0-G1 phase based on concentration levels (Fig. 1F, G). VER decreased the levels of proteins associated with cell growth, including PCNA, c-Myc, and cyclin D1 (Fig. 1H, I). The immunofluorescence staining results revealed a substantial decrease in PCNA expression in 143B cells following VER treatment (Fig. 1J). Based on the above, VER could inhibit proliferation and cause G0-G1 phase arrest in OS cells.

### Veratramine inhibits the migration and invasion of human OS cells

**Figure 2:**
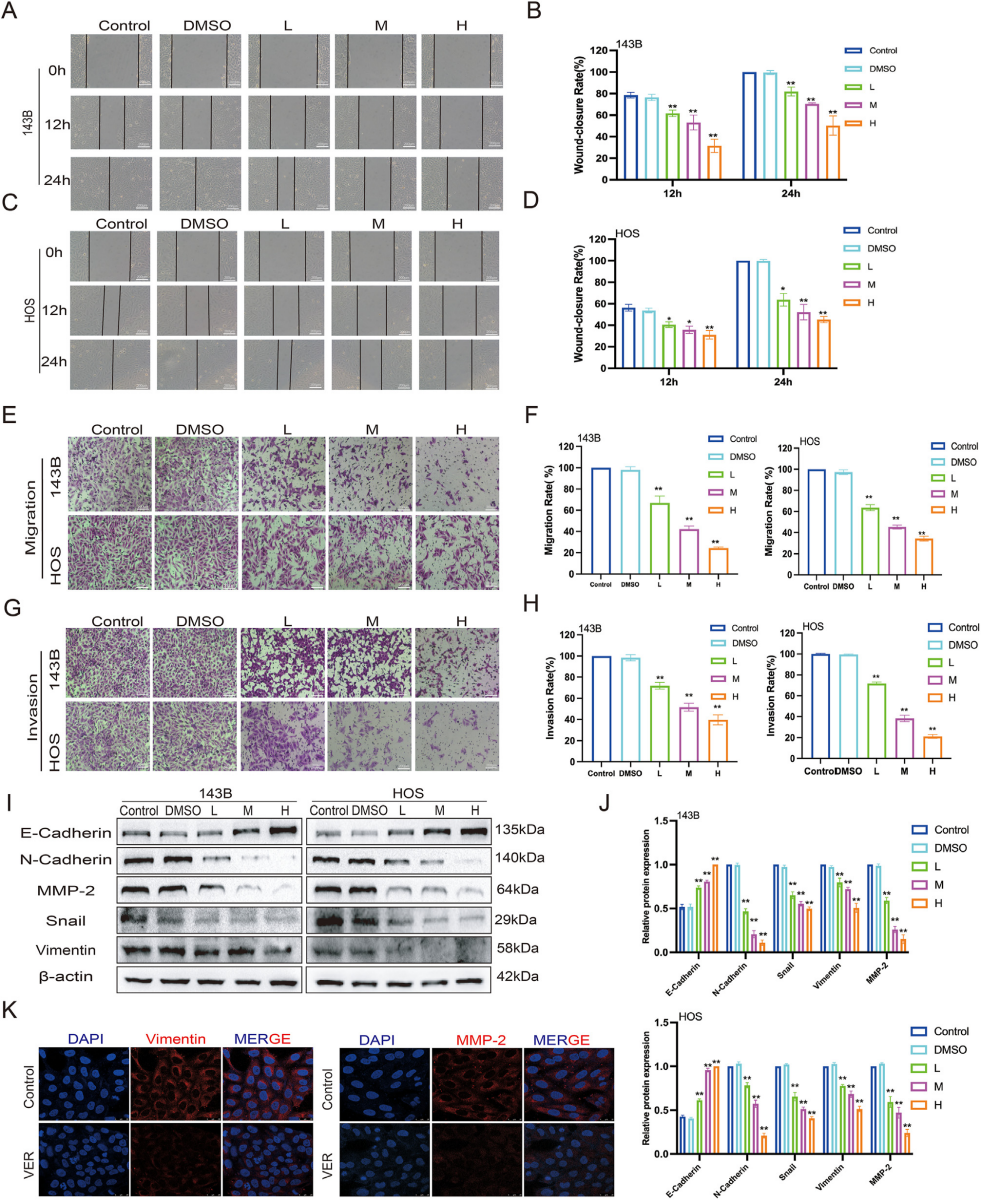
Veratramine suppresses the movement and penetration of human osteosarcoma cells. **(A**–**D)** The effects of VER on the migration abilities of 143B and HOS cells were detected by wound healing test. **(E**–**H)** The effects of VER on the migration and invasive abilities of 143B and HOS cells were detected by transwell assay. **(I, J)** The protein levels of matrix metallopeptidases (MMPs) and epithelial-mesenchymal transition-related molecules in 143B and HOS were tested by Western blot. **(K)** Vimentin and MMP-2 in the cytoplasm of 143B were detected by immunofluorescence test, with vimentin and MMP2 labeled with Cy-3 in red and the nucleus labeled in blue with DAPI. Results are presented as mean ± standard deviation. Statistical significance is indicated by ∗*p* < 0.05 and ∗∗*p* < 0.01 versus the control group.

To explore the impact of VER on the migration and invasion of human OS cells, we observed a notable decrease in the wound healing rate after administering VER (Fig. 2A–D), suggesting a substantial suppression of OS cell migration. VER treatment reduced the number of migratory cells via the upper chamber, suggesting its capacity to inhibit OS cell migration (Fig. 2E, F). Furthermore, we discovered that VER significantly reduced the invasive ability of OS cells, as shown by fewer cells invading through Matrigel-coated membranes (Fig. 2G, H). EMT and matrix breakdown are critical stages in tumor migration and invasion.^23^ Therefore, we used Western blot to determine the protein levels of EMT markers and MMPs. The findings indicated that VER markedly suppressed the protein concentrations of the pro-EMT markers N-cadherin, Snail, and vimentin. In addition, the protein level of extracellular matrix degrader MMP2 was dramatically reduced (Fig. 2I, J). The immunofluorescence staining results revealed a substantial decrease in vimentin and MMP2 expression in 143B cells following VER administration (Fig. 2K). These findings suggest that VER could hinder the migration and invasion of OS cells.

### Veratramine induces apoptosis in human OS cells

**Figure 3:**
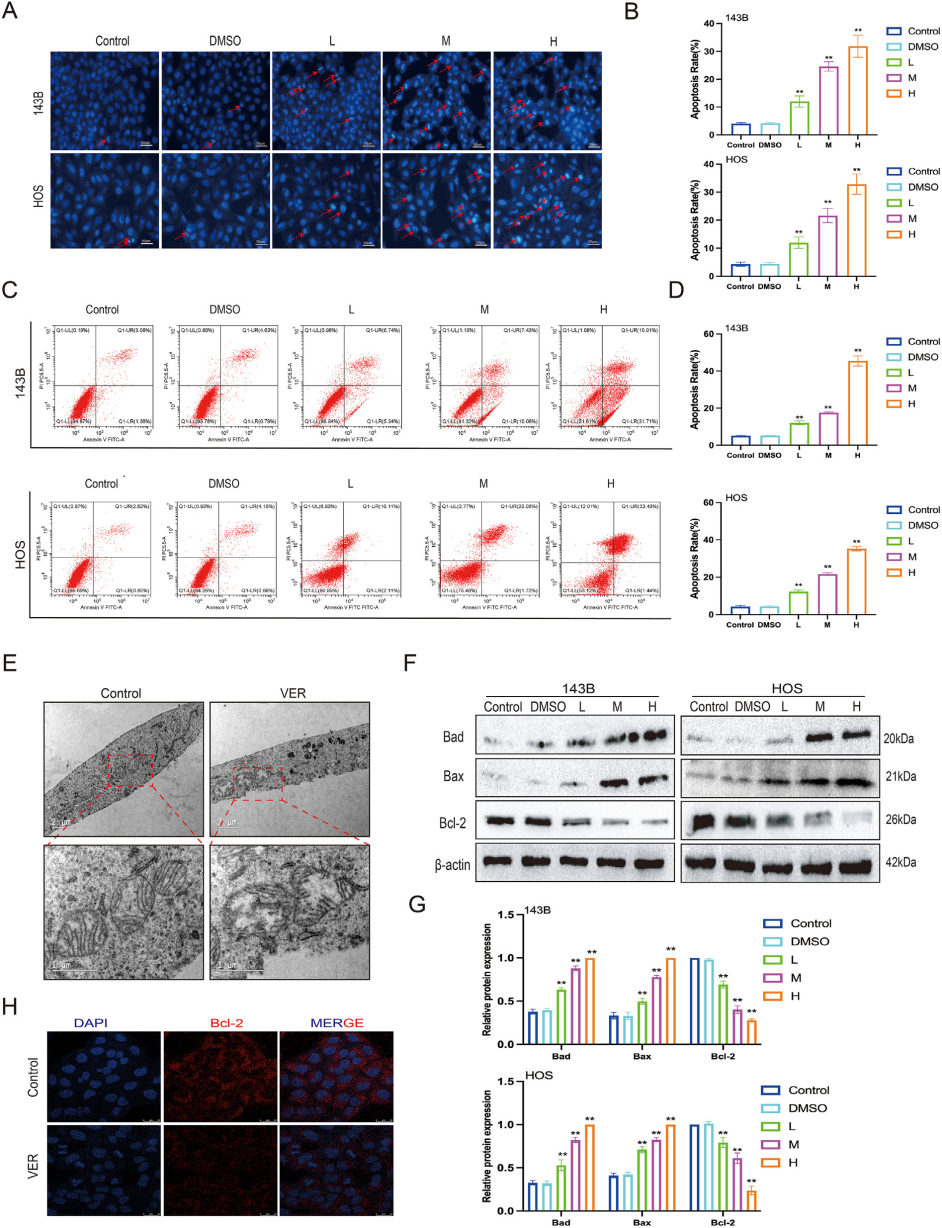
Veratramine promotes apoptosis of human osteosarcoma cells. **(A**–**D)** The effects of VER on the apoptosis of 143B and HOS cells were detected by Hoechst 33258 staining assay (A, B) and flow cytometry (C, D). **(E)** Representative transmission electron microscopy images of 143B cells in the control and VER group. **(F, G)** The protein levels of apoptosis-related molecules in 143B and HOS were tested by Western blot. **(H)** Detection of Bcl2 expression of 143B by immunofluorescence, labeled Bcl2 with Cy-3 (red) and labeled nucleus with DAPI (blue). Results are presented as mean ± standard deviation. Statistical significance is indicated by ∗*p* < 0.05 and ∗∗*p* < 0.01 versus the control group.

It was observed that a 24-h exposure to varying doses of VER increased the number of apoptotic cells as the VER concentration rose in comparison to the control group. This was indicated by Hoechst 33258 staining (Fig. 3A, B). We then investigated the impact of VER on apoptosis in OS cells using flow cytometry. Higher VER concentrations increased the overall early and late apoptotic fractions of OS cells (Fig. 3C, D). Transmission electron microscopy indicated the creation of numerous vesicles on the cell membrane, mitochondrial enlargement, and loss of mitochondrial cristae, implying that VER administration may induce apoptosis in 143B cells Fig. 3E). Similarly, Western blot analysis revealed that VER raised Bax and Bad expression while decreasing Bcl-2 expression (Fig. 3F, G). The immunofluorescence staining results revealed a substantial decrease in BCL2 expression in 143B cells following VER treatment (Fig. 3H). The findings indicate that VER triggers cell apoptosis in OS cells.

### Network pharmacology analysis of Veratramine's mechanism of anti-tumor effects in OS

**Figure 4:**
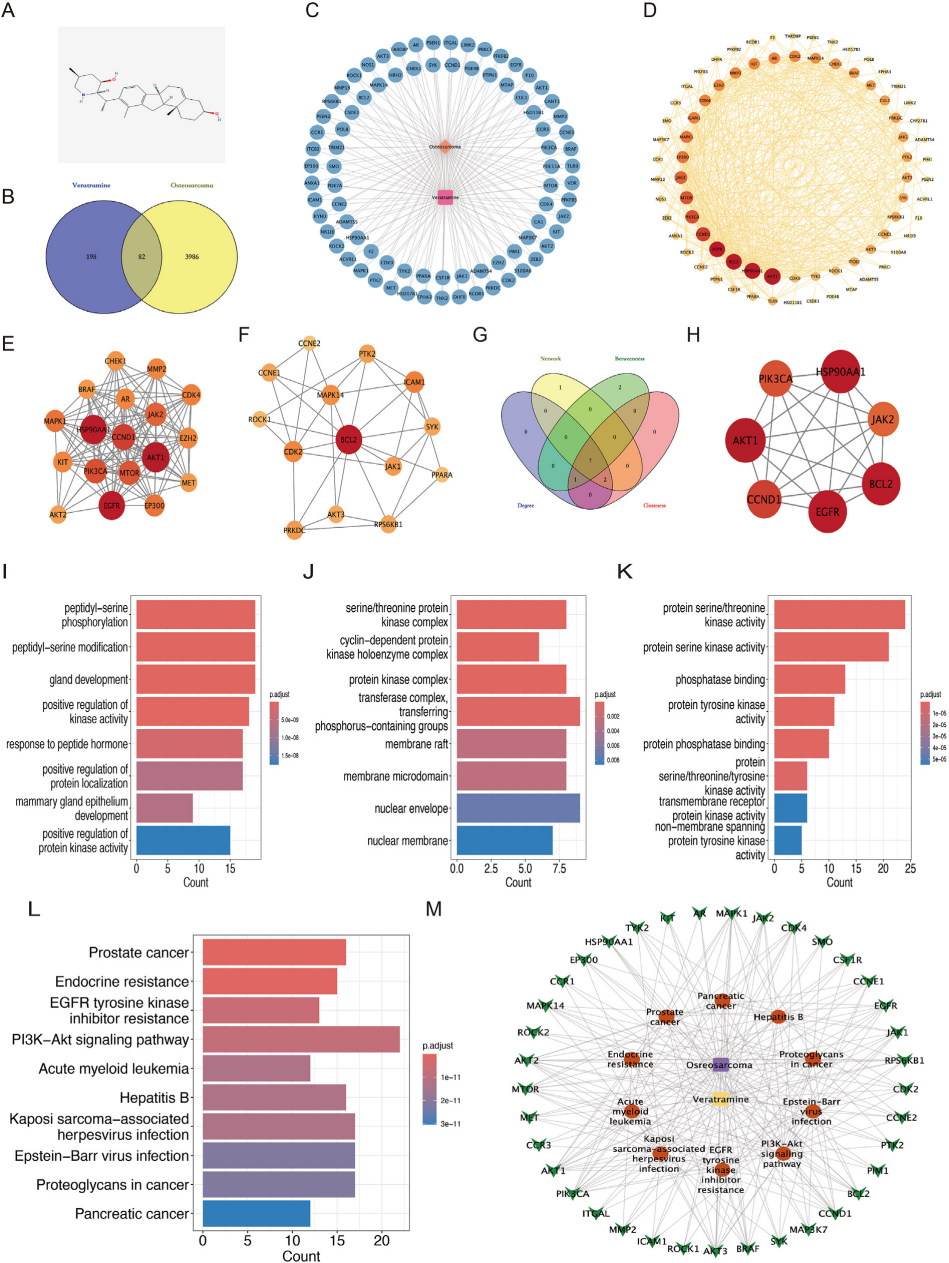
Analysis of the mechanism of veratramine on osteosarcoma based on network pharmacology. **(A)** The chemical structure of VER. **(B)** Venn diagram of the target of VER and the target of OS. **(C)** Compound-target network of VER. Pink represents VER, blue represents 82 common targets, and orange represents OS. **(D)** The protein interaction network diagram. The size, color, and depth changes of nodes represent the size of the degree value. The core targets were ranked based on protein–protein interaction analysis. **(E, F)** Two clusters based on cluster analysis using the MCODE plug-in. **(G, H)** Venn diagram of four algorithms employed to screen seven core candidate targets (G) and protein–protein interaction network of the seven core candidate targets (H). **(I–K)** Top 10 biological process (BP) terms, cellular component (CC) terms, and molecular function (MF) terms of GO enrichment analysis. **(L)** The bar plot of the top 10 pathways based on KEGG enrichment analysis. **(M)** The “drug-target-pathway-disease” network diagram.

Figure 4A illustrates the chemical composition of VER, with the molecular formula (C27H39NO2) and a molecular weight of 409.6 g/mol. The Pharmmapper and Swiss Target Prediction databases were used to identify 280 VER-associated targets. In addition, searches in OMIM, DisGeNET, TTD, and GeneCards yielded 4068 OS-related targets. By utilizing the Venny 2.1 web application (https://bioinfogp.cnb.csic.es/tools/venny/), a total of 82 shared drug-disease targets were discovered by overlapping 280 drug targets with 4068 disease targets (Fig. 4B). A network diagram labeled “Disease-Target-Component” was generated with Cytoscape 3.9.1 (Fig. 4C), featuring VER highlighted in pink, 82 shared targets marked in blue, and the Osteosarcoma depicted in orange. The 82 common targets were then evaluated for protein–protein interactions using the STRING database, and a protein–protein interaction network diagram was created in Cytoscape (Fig. 4D), with node depth representing degree values. The MCODE module detected two gene clusters (Fig. 4E, F). The CytoNCA plugin was used to filter core candidate targets using Degree, Network, Betweenness, and Closeness algorithms, yielding seven core candidate targets (Fig. 4G), including AKT1, CCND1, EGFR, BCL2, JAK2, HSP90AA1, and PI3KCA, as shown in Figure 4H.

To understand how VER works in OS, we utilized the R package clusterProfiler to perform GO and KEGG enrichment analyses on the 82 common targets. The GO analysis identified 1351 biological process elements (Fig. 4I), such as the regulation of kinase activity, peptidyl serine phosphorylation and peptidyl-serine modification. The examination of cellular components identified 43 entities (Fig. 4J), such as the serine/threonine-protein kinase assembly, the cyclin-dependent protein kinase holoenzyme group, and the membrane raft. The analysis of molecular functions comprised 67 items (Fig. 4K), featuring activities such as protein serine/threonine kinase, phosphatase binding, and protein tyrosine kinase. KEGG analysis indicated an abundance in pathways such as the PI3K/AKT signaling cascade, acute myeloid leukemia, hepatitis B, and resistance to EGFR tyrosine kinase inhibitors. The top ten relevant paths were depicted (Fig. 4L). The incorporation of shared target genes and the ten most prevalent KEGG pathways into Cytoscape 3.9.1 resulted in the generation of a “Drug-Target-Pathway-Disease” network diagram (Fig. 4M), which illuminated the potential mechanisms by which VER may exert its effects against OS. In conclusion, VER's pharmacological effects involve close synergistic linkages and substantial binding activities among its targets, demonstrating its multifarious pharmacological properties, multi-target effects, and involvement in various signaling cascades against OS. These anticipated molecular processes provide a theoretical foundation for future fundamental pharmacological research on VER.

### Veratramine's molecular docking with key target

**Figure 5:**
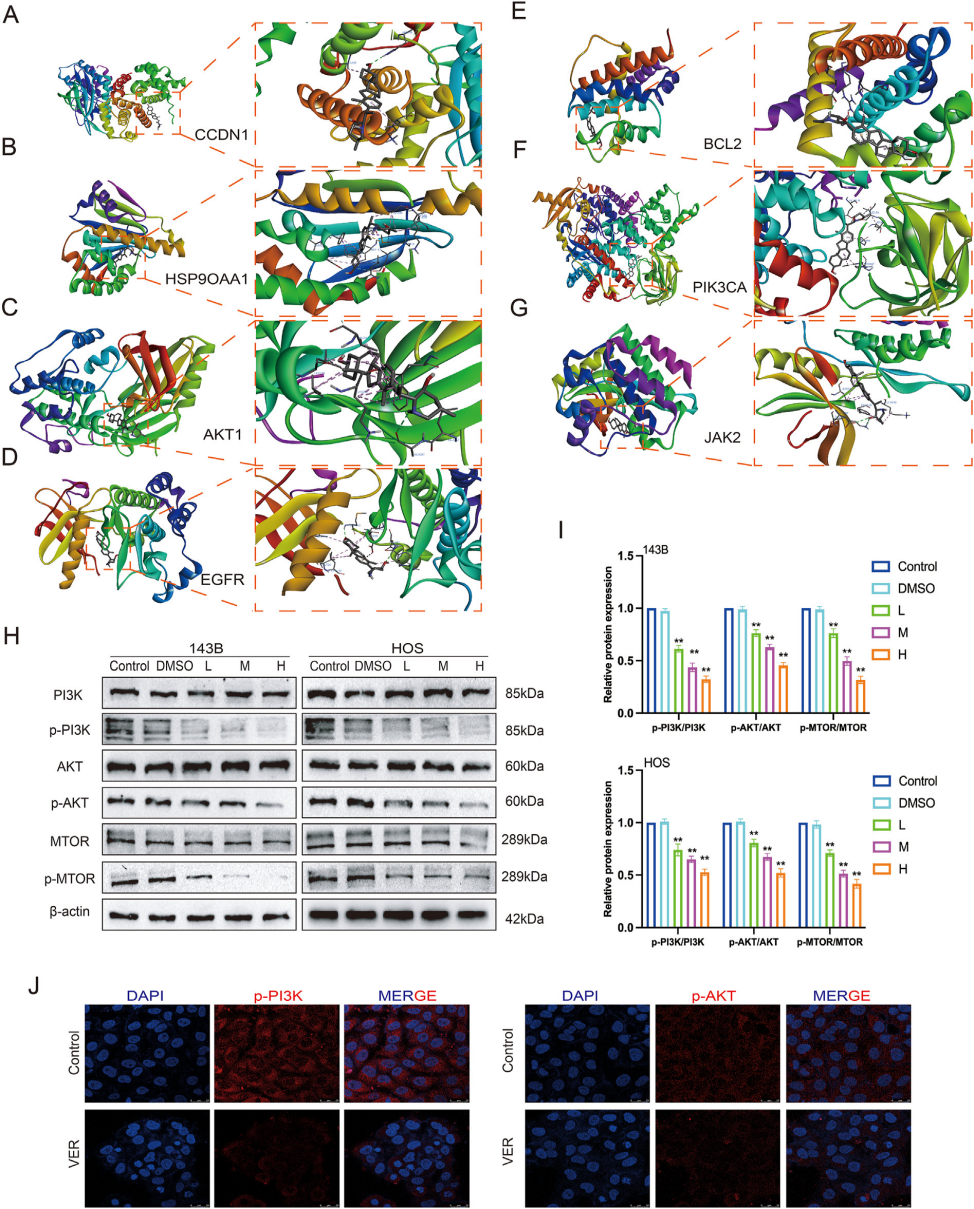
Molecular docking and PI3K/AKT signaling pathway validation. **(A**–**G)** Molecular docking of VER and AKT1, HSP90AA1, BCL2, EGFR, CCND1, JAK2, and PI3KCA. **(H, I)** The protein levels of PI3K/AKT-related molecules in 143B and HOS were tested by Western blot. **(J)** PI3K and AKT in the cytoplasm of 143B were detected by immunofluorescence test, with PI3K and AKT labeled in red with CY5 and the nucleus labeled in blue with DAPI. Results are presented as mean ± standard deviation. Statistical significance is indicated by ∗*p* < 0.05 and ∗∗*p* < 0.01 versus the control group.

To determine if VER exerted its therapeutic benefits in OS via important pathways mentioned above, we performed preliminary validation using molecular docking. VER was docked with core target proteins selected by a combination of four methods in PyRX software. Figure 5A–G shows how molecular docking results were visualized and analyzed using Discovery Studio. VER had the lowest binding energies with AKT1, HSP90AA1, BCL2, EGFR, CCND1, JAK2, and PI3KCA, with values of −10.8, −10.8, −7.6, −8.9, −7.6, −9, and −8.7 kJ/mol, respectively (Table S2). A lower binding energy means that the ligand and receptor are more likely to interact. Each of these primary targets exhibited binding energies below −5.0 kJ/mol, suggesting a robust interaction between the ligand and receptor. These key targets are largely linked to the PI3K/AKT pathway. These data indicate that VER likely suppresses the PI3K/AKT signaling cascade in OS cells by binding tightly to core target proteins.

### Veratramine suppresses OS cell growth via the PI3K/AKT signaling pathway

**Figure 6:**
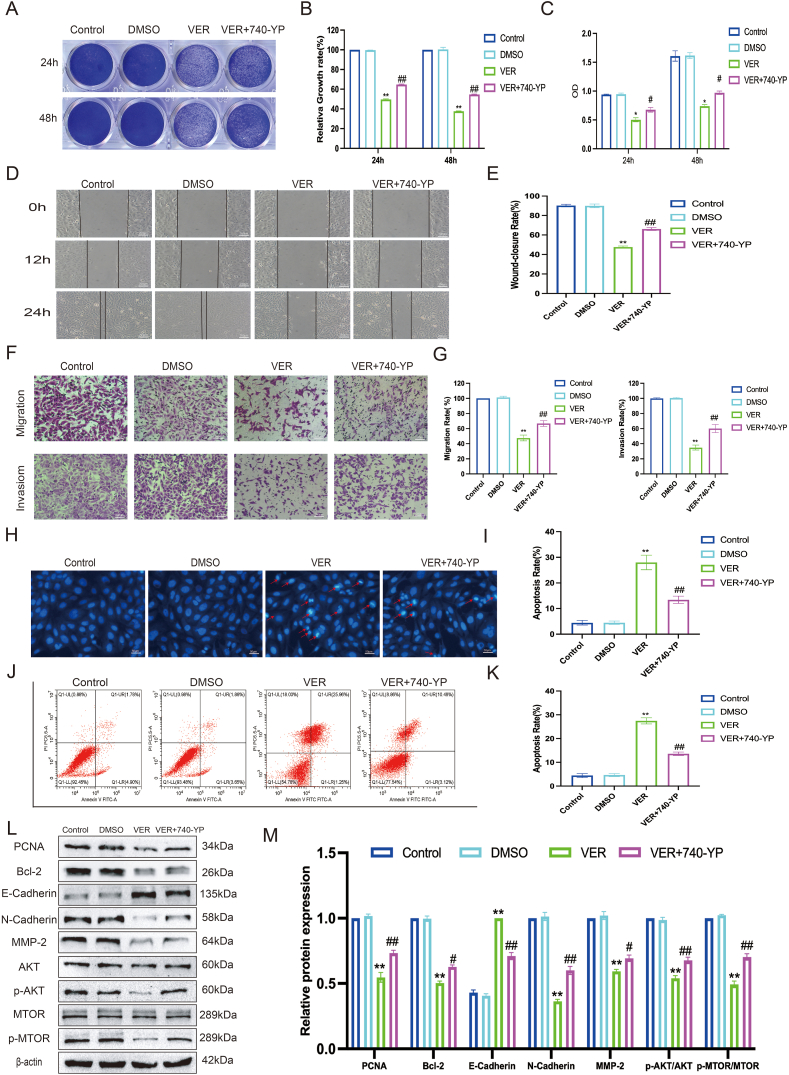
Veratramine inhibits osteosarcoma by regulating the PI3K/AKT signaling pathway. **(A**–**C)** The effects of VER on the proliferation of OS cells in the presence of 740-YP were detected by crystal violet staining (A, B) and CCK8 assay (C). **(D**–**G)** The effects of VER on the migration and invasion of OS cells in the presence of 740-YP were detected by wound healing test (D, E) and transwell assay (F, G). **(H)** The effects of VER on the apoptosis of OS cells in the presence of 740-YP were detected by Hoechst 33258 staining assay (H,I) and flow cytometry (J,K). **(L, M)** The protein levels of the proliferation, apoptosis, migration, and invasion related molecules in OS cells were tested by Western blot. Results are presented as mean ± standard deviation. Statistical significance is indicated by ∗*p* < 0.05 and ∗∗*p* < 0.01 versus the control group and ^#^*p* < 0.05 and ^##^*p* < 0.01 versus the VER group.

To aid in the pharmacological network analysis of VER's potential effects on OS cells, we examined alterations in key proteins within the PI3K/AKT signaling pathway. The Western blot analysis demonstrated that treatment with VER markedly decreased the levels of p-PI3K/PI3K, p-AKT/AKT, and p-MTOR/MTOR compared with the control group (Fig. 5H, I). Immunofluorescence staining revealed a significant decrease in p-PI3k and p-AKT levels in 143B cells following VER administration (Fig. 5J). We examined how VER affected the growth of human OS cells via the PI3K/AKT signaling pathway by treating 143B cells with 10 μM of 740-YP, an activator of the PI3K/AKT pathway, and 40 μM of VER. We investigated if this activator might influence VER's inhibitory effects on OS cells. At first, we found that 740-YP somewhat reinstated VER's suppression of OS cell growth, as assessed by crystal violet staining and the CCK-8 assay, when compared with the VER treatment group (Fig. 6A–C). Subsequently, we discovered that 740-YP partially restored VER's suppression of OS cell migration and invasion (measured by wound healing and transwell assay) (Fig. 6D–G). Furthermore, we discovered that 740-YP partially reversed VER's increase in OS cell death (as evidenced by Hoechst 33258 staining and flow cytometry) (Fig. 6H–K). Our study demonstrated that 740-YP treatment led to higher levels of PCNA and C-Myc related to proliferation, as well as Bcl-2 related to apoptosis. Additionally, in 143B cells treated with VER, 740-YP treatment enhanced the expression of proteins linked to migration and invasion, such as N-Cadherin, and MMP2, while concurrently decreasing E-Cadherin expression, as shown by Western blot analysis (Fig. 6L, M). In conclusion, VER likely inhibits OS growth by regulating the PI3K/AKT signaling pathway.

### Veratramine has a low toxicity *in vivo*

**Figure 7:**
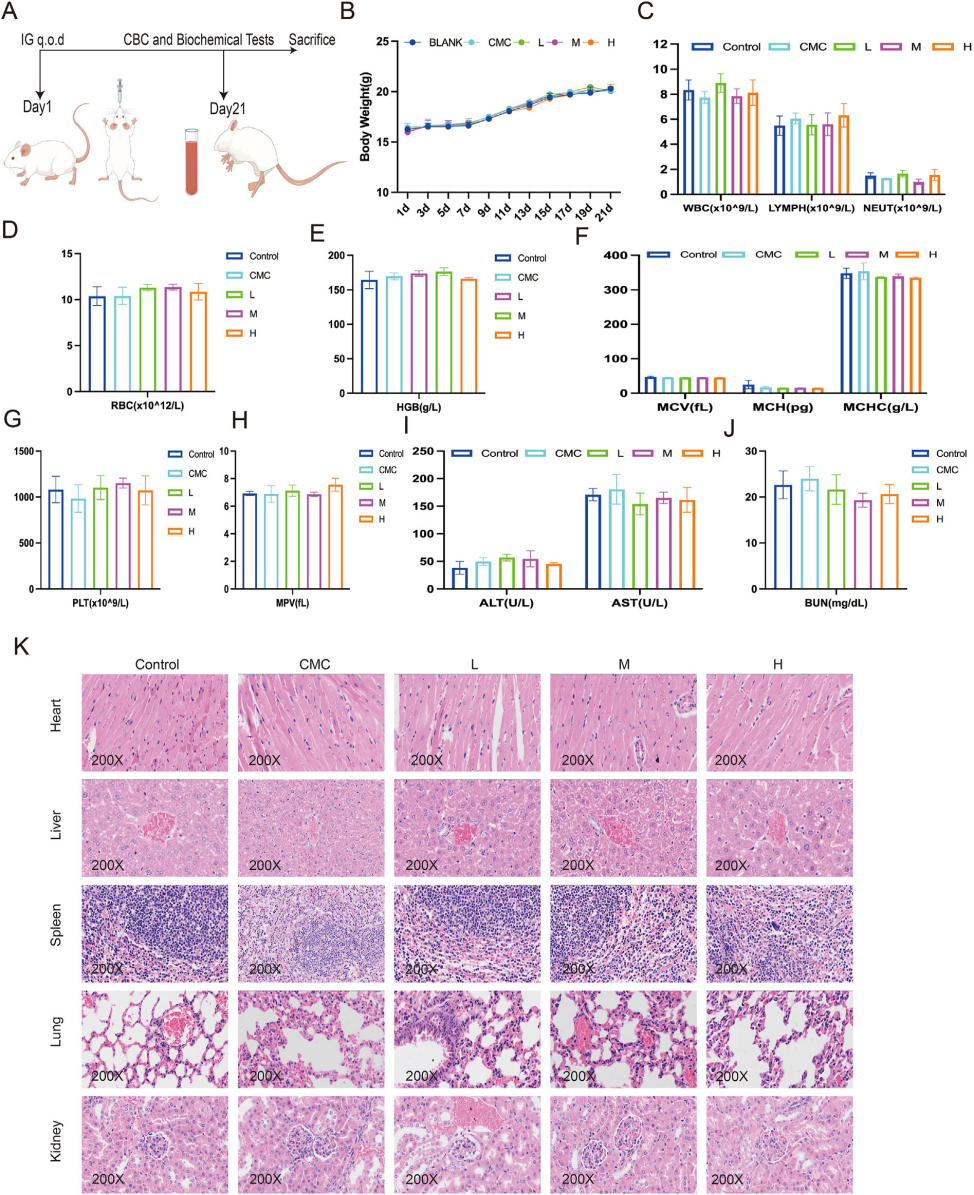
Veratramine has no significant toxicity *in vivo*. **(A)** Schematic illustration of the *in vivo* toxicity measurement of VER in BALB/c mice. **(B)** The body weight of BALB/c mice. **(C)** The effects of VER on total white blood cell (WBC), lymphocyte, and neutrophil counts. **(D, E)** The effects of VER on red blood cell (RBC) count and hemoglobin (HGB) level. **(F)** The effects of VER on mean cell volume (MCV), mean corpuscular hemoglobin (MCH), and mean corpuscular hemoglobin concentration (MCHC) levels. **(G, H)** The effects of VER on platelet (PLT) count and mean platelet volume (MPV) level. **(I)** The effects of VER on the levels of serum alanine transaminase (ALT) and aspartate aminotransferase (AST). **(J)** The effects of VER on the level of blood urea nitrogen (BUN). **(K)** The effects of VER on the histological structure of the heart, liver, spleen, lungs, and kidneys (hematoxylin-eosin staining). Data are shown as mean ± standard deviation from three independent experiments. Statistical significance is indicated by ∗*p* < 0.05 and ∗∗*p* < 0.01 versus the control group.

Toxicity studies were conducted in immunocompetent mice to ascertain the safety of VER, as demonstrated in Figure 7A. VER was first administered orally to healthy mice in a series of doses, followed by an *in vivo* assessment of its toxicity. We found no significant variations in body weight across the groups (Fig. 7B). Additionally, our analysis revealed no notable alterations in blood metrics including white blood cells, lymphocytes, neutrophils, red blood cells, hemoglobin, mean corpuscular volume, mean corpuscular hemoglobin, mean corpuscular hemoglobin concentration, platelets, and mean platelet volume (Fig. 7C–H). This implies that VER has low to no hematological toxicity.

Additionally, there were no notable differences in the serum concentrations of liver damage indicators ALT and AST, or the kidney damage marker BUN, among the groups (Fig. 7I, J). A histopathological analysis with hematoxylin-eosin staining indicated no cellular edema, tissue damage, or major pathological alterations in the heart, liver, kidneys, spleen, or lungs after VER administration (Fig. 7K). These data collectively confirm VER's good safety profile *in vivo*, laying the groundwork for future experimental trials in OS animal models.

### Veratramine suppresses the growth of OS cells *in vivo*

**Figure 8:**
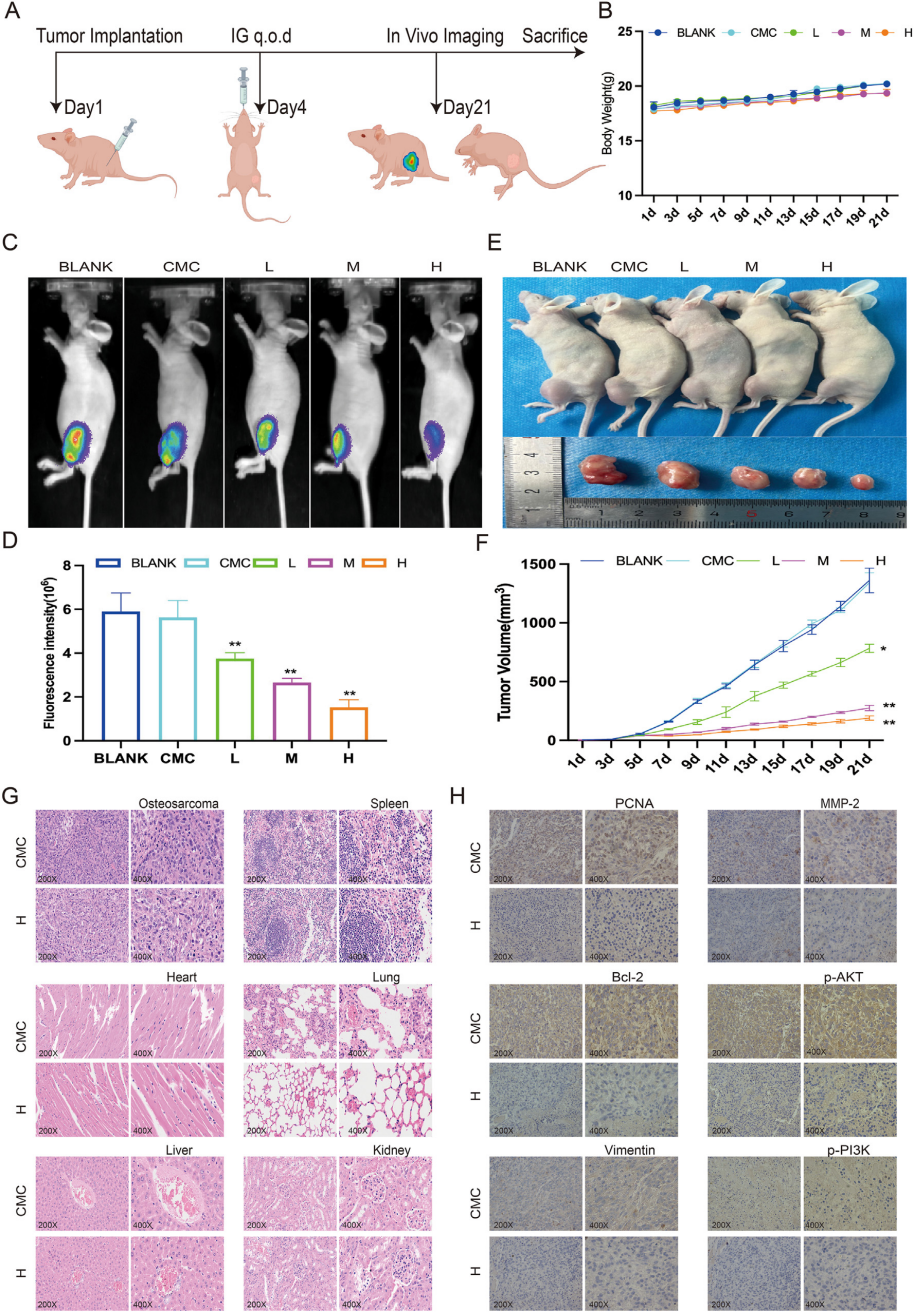
Veratramine inhibits the growth and metastasis of osteosarcoma cells *in vivo*. **(A)** Schematic illustration of the *in vivo* anti-tumor effects of VER in BALB/c nude mice. **(B)** The body weight of nude mice. **(C)** The *in vivo* fluorescence images of 143B-fluc tumor-bearing mice treated with different drug groups on day 21. **(D)** Statistical analysis of relative fluorescence intensity. **(E)** The representative photo of tumors. **(F)** The effects of VER on tumor volume. **(G)** The effects of VER on tumors, lungs, heart, liver, spleen, and kidneys were detected by hematoxylin-eosin staining. **(H)** PCNA, Bcl-2, vimentin, MMP-2, p-PI3K, and p-AKT were detected by immunohistochemistry. Statistical significance is indicated by ∗*p* < 0.05 and ∗∗*p* < 0.01 versus the control group.

To see if VER's *in vivo* effects were consistent with its *in vitro* anti-tumor activity, we created a nude mouse model of OS in the tibia and administered different amounts of VER via gavage, as shown in Figure 8A. The findings indicated that body weight remained consistent among the different groups of mice (Fig. 8B). Real-time imaging revealed a significantly diminished fluorescent signal at the tumor location in treated subjects compared with the control group, suggesting a decrease in tumor cell activity and growth (Fig. 8C, D). Similarly, the VER therapy group had significantly smaller tumors than the control group (Fig. 8E, F). Hematoxylin-eosin staining of tumor tissues revealed that the high-concentration VER treatment group had considerably lower tumor cell density and features such as nuclear condensation and fragmentation than the control group. Hematoxylin-eosin staining of lung tissues revealed considerably lower metastatic foci in the high-concentration VER therapy group, as well as cleaner lung tissue structure. Hematoxylin-eosin staining on cardiac, hepatic, splenic, and renal tissues revealed no notable morphological variations among the groups (Fig. 8G).

Immunohistochemical analysis showed that tumor tissues treated with high doses of VER exhibited reduced levels of PCNA, Bcl-2, vimentin, MMP-2, p-AKT, and p-PI3K compared with the control group (Fig. 8H). These data show VER's ability to limit the growth of OS, ease symptoms of lung metastasis *in vivo*, and validate its prospective therapeutic efficacy against OS.

## Discussion

OS mainly affects children and teenagers. Despite advances in treatment procedures, long-term efficacy is still limited. Chemotherapy is the primary treatment for OS, although its efficiency is frequently hampered by variables such as tumor spread, drug resistance, and damage to normal organs.^5^ To improve treatment outcomes and patient prognosis, researchers have prioritized the development of novel, efficient, and low-toxicity medications.

VER has been demonstrated to be anti-cancer in different cancers, including glioblastoma, prostate, and liver cancer.^11^, ^12^, ^13^ In this work, we looked at the anti-tumor effects of VER in OS. Our research found that VER halted cell proliferation by causing G0/G1 phase arrest and diminished the metastatic potential of human OS cells by altering EMT markers. Additionally, VER administration caused apoptosis in OS cells. This suppressive effect might be associated with the PI3K/AKT/mTOR signaling pathway. Furthermore, Its safety has also been confirmed *in vivo*.

In this investigation, we discovered that VER suppressed OS cell viability, reduced clonogenic ability, decreased PCNA and C-Myc protein levels, and slowed the growth of OS *in vivo*. These results indicate that VER may have a potent anti-proliferative effect on OS cells. Uncontrolled cell development is a characteristic of carcinogenesis, defined by proliferation abnormalities.^24^ Regulating the cell cycle is crucial to limiting the aberrant growth of cancer cells.^25^ Inducing tumor cell cycle arrest is an effective method, and common clinical chemotherapeutic agents such as gemcitabine, cisplatin, and vinorelbine exhibit anti-tumor efficacy by breaking the cell cycle.^26^, ^27^, ^28^ In this study, we found that administering VER led to G0/G1 phase cell cycle arrest in 143B and HOS cell lines, subsequently reducing the expression of cyclin D1 associated with the G1 phase. Cyclin D1 is an essential protein that regulates the cell cycle, particularly during the shift from the G1 phase to the S phase. The excessive expression or abnormal activation of it is linked to the development and advancement of various cancers.^29^ Interestingly, in a study of VER in glioma, it also caused G0/G1 phase cell cycle arrest in three glioma cell lines by preventing the development of the cyclin D-CDK4/CDK6-associated complex.^11^

The effectiveness of cancer therapies and the outlook for patients is significantly influenced by the capability of tumor cells to move from primary tissues to remote secondary locations.^30^ This process involves EMT, which is a biological process in which tumor epithelial cells change into mesenchymal tissue.^23^ In this phase, stationary epithelial cells shed their polarity and intercellular bonds, evolving into mobile and invasive mesenchymal cells.^31^ In this study, wound healing and transwell studies demonstrated that VER could inhibit tumor migration and invasion. Western blot analysis demonstrated that VER inhibited the EMT process. E-cadherin prevents tumor cell migration and invasion by preserving cell–cell adhesion and structural integrity, whereas decreased expression enhances these processes. N-cadherin produces the opposite effect.^32^ Snail is a crucial transcription factor that attaches to the promoter area of the E-cadherin gene, inhibiting its transcription while promoting the expression of mesenchymal markers such as vimentin, fibronectin, and N-cadherin.

Additionally, extracellular matrix breakdown is required for tumor cells to move and infiltrate the basement membrane. Studies have demonstrated that MMPs have a critical role in degrading the extracellular matrix, degrading the main components of the extracellular matrix, and creating paths for cell migration and invasion.^33^ Our research indicates that VER could inhibit the migration and invasion of OS cells by reversing the EMT and reducing the MMPs. To conclude, VER can inhibit the migration and invasion of OS cells.

Apoptosis dysregulation is another characteristic of malignancies and influences tumor treatment resistance.^24^ Currently, targeting apoptotic pathways has become a promising therapeutic anti-cancer therapy.^34^ Apoptosis in a cell can be initiated through two separate mechanisms: the intrinsic mitochondrial (BCL-2) pathway and the extrinsic death receptor pathway. The BCL-2 protein family regulates the intrinsic route, and important genes in apoptosis include Bcl-2, Bax, and Bad. Apoptotic factors like cytochrome C and Smac/DIABLO are released when Bad binds tightly to the anti-apoptotic protein BCL2, releasing Bax and Bak.^35^ This process of oligomerization on the external mitochondrial membrane leads to mitochondrial outer membrane permeability, subsequently triggering the caspase cascade and intensifying the apoptotic signal.^36^ Our study revealed that VER could trigger cell death in OS by decreasing the levels of proteins that prevent apoptosis and increasing those that promote it. Immunofluorescence and Hoechst staining revealed chromatin condensation and nuclear disintegration. Additionally, transmission electron microscopy revealed apoptosis-related morphological alterations in the mitochondria of OS cells treated with VER. In studies of VER's anti-cancer effect on liver cancer and glioma, tumor cells undergo apoptosis. We believe this phenomenon is due to VER acting as an AP-1 modulator, influencing various biological activities such as cell growth and programmed cell death.^14^^,^^37^ Overall, VER increases apoptosis in OS cells and may be a pan-tumor apoptosis inducer.

In this study, our findings demonstrate that VER not only exhibits significant inhibitory effects on OS cells but also merits further exploration of its applicability in other cancer types. As a natural alkaloid, VER has gained increasing attention in recent years, showing promising anti-cancer potential across various malignancies. Existing research indicates that VER effectively inhibits cell proliferation in liver cancer, glioma, and prostate cancer, and it also offers protective effects against ultraviolet-induced skin carcinogenesis.^11^, ^12^, ^13^, ^14^^,^^38^

Specifically, studies on liver cancer have revealed that VER can effectively suppress the proliferation of liver cancer cells and induce autophagy-mediated apoptosis, primarily through the inhibition of the PI3K/Akt signaling pathway. Furthermore, its application in glioma and prostate cancer has demonstrated a significant ability to inhibit cell migration and invasion. Recent research utilizing organoid models has underscored the critical potential of VER in modulating drug resistance in liver cancer.^22^ Drug resistance remains a major obstacle to effective treatment; literature suggests that c-Jun acts as a key mediator of lenvatinib resistance.^39^ VER has been shown to effectively inhibit c-Jun activity, thereby countering resistance mechanisms. This finding provides new insights into the future application of VER in liver cancer treatment, highlighting its unique advantages in addressing drug resistance.

Current network pharmacology employs public data to identify drug targets and critical targets using topological analysis, followed by molecular docking to analyze drug–target interactions.^40^^,^^41^ In this investigation, we identified 4068 OS targets and 280 VER targets, and 82 shared drug-disease targets were discovered by overlapping. By constructing a protein–protein interaction network and performing GO and KEGG pathway enrichment analyses, we discovered seven key targets of VER in the treatment of OS. These targets include AKT1, CCND1, EGFR, BCL2, JAK2, HSP90AA1, and PI3KCA. PI3KCA (phosphatidylinositol 3-kinase catalytic subunit alpha) is a key player in the PI3K/Akt signaling cascade. In OS, aberrant PI3KCA activation increases tumor cell proliferation and survival, while mutations are related to tumor invasiveness and medication resistance. AKT1 (protein kinase B1), an essential downstream component of the PI3K/Akt signaling pathway, promotes cell growth and inhibits programmed cell death in OS, leading to tumor development and resistance to treatment.^17^^,^^18^ Cyclin D1 (CCND1) plays a role in the transition from the G1 phase to the S phase of the cell cycle. Its overexpression in OS enhances tumor cell proliferation and is linked to tumor aggressiveness and a poor prognosis.^42^ EGFR (epidermal growth factor receptor) aberrant activation in OS enhances cell proliferation and migration, and its targeted therapy has potential efficacy in OS treatment.^43^ BCL2, a protein that prevents cell death, supports the survival of OScells by inhibiting apoptosis. Elevated levels of BCL2 have been linked to the aggressiveness of OS and unfavorable outcomes.^35^ JAK2 (tyrosine kinase 2) promotes tumor growth and treatment response in OS by regulating cell proliferation and immune response.^44^ HSP90AA1 (heat shock protein 90 alpha 1) regulates numerous oncogenic proteins involved in OS, and its overexpression enhances tumor cell survival and therapy resistance.^45^ Abnormal expression and function of these targets are typically related to tumor development and a poor prognosis. Targeting these molecules holds the potential for improving tumor patient prognosis and treatment results.

Analysis of the KEGG pathway indicated that the PI3K-AKT signaling pathway could be the main mechanism through which VER achieves its anti-OS effects. Numerous studies indicate that triggering the downstream target genes of this pathway is crucial for OS progression. Growth factors and cytokines activate PI3K in cells, causing it to phosphorylate and attach to the Akt domain. This allows Akt to move from the cytoplasm to the cell membrane, where it changes shape and becomes fully activated through phosphorylation at the Ser473/Thr308 locations, ultimately controlling the function of its downstream targets.^17^^,^^18^ Our research shows a significant decrease in the proteins associated with the PI3K/AKT signaling pathway in OS cells exposed toVER. To validate this discovery, we examined if the PI3K/AKT pathway activator 740-YP could counteract the suppressive impact of VER on OS. Multiple results found that 740-YP partially reversed the inhibitory effects of VER on OS cells.

Based on the above results, we can suggest that VER may exert its inhibitory effects on OS by targeting the PI3K pathway. However, whether it specifically targets this pathway requires further investigation in the future. We also noted studies indicating that VER is a potent natural regulator of AP-1, capable of selectively binding to specific DNA sequences of AP-1 target sites (TRE 5′-TGACTCA-3′).^14^ The AP-1 transcription factor is a heterodimeric protein complex mainly composed of different proteins from the c-Fos, c-Jun, ATF, and MAF families, and is closely associated with the regulation of cellular processes such as proliferation, apoptosis, senescence, differentiation, migration, immunity, and inflammation.^37^ VER has been shown to effectively inhibit the activation of AP-1 in a dose-dependent manner.^14^ These findings provide a supportive background for the potential application of VER as a broad-spectrum anti-tumor agent. Moreover, there may be crosstalk between AP-1 and the PI3K signaling pathway. Numerous studies have indicated that PI3K signaling can influence the activation of AP-1, triggering a cascade of responses.^46^, ^47^, ^48^ Through the KDM3A-dependent AP-1 transcriptional mechanism, activation of the PI3K signaling pathway promotes liver tumorigenesis, with KDM3A playing a crucial bridging role in this process.^46^ In another study on cervical cancer, it was shown that AP-1 could affect the PI3K signaling pathway by regulating HPV E7-mediated NCAPH expression, thereby enhancing the proliferation and survival of cervical cancer cells. This suggests a complex regulatory relationship between AP-1 and PI3K. Such crosstalk may play a significant role in the progression of OS and warrants further investigation in future studies.^49^ In particular, we aim to explore the potential effects of VER on the PI3K/AKT signaling pathway and its relationship with the AP-1 transcription factor. Understanding how VER may modulate these key molecular interactions could provide new insights into its therapeutic potential in OS treatment.

It should be mentioned that safety is always the top priority when it comes to *in vivo* medicinal uses. Previous research has demonstrated that VER can oppose sodium channels, reduce blood pressure, and relieve neuropathic pain, establishing the groundwork for potential *in vivo* applications.^50^^,^^51^ Similarly, our *in vivo* study results revealed that mice treated with VER did not lose substantial weight. The hematological values remained normal throughout the trial. Liver injury indicators (ALT, AST) and renal injury marker (BUN) were normal. Hematoxylin-eosin staining revealed no significant pathological alterations in major organs, confirming the safety of VER in mice. Earlier studies have documented the sex-specific pharmacokinetics of VER in rodents.^52^ We conducted our experiments with female mice. In future investigations, we will investigate how gender impacts VER's anti-cancer activity to provide the groundwork for its therapeutic usage. Overall, these findings indicate that VER is both effective and safe against OS.

However, several limitations need to be addressed. First, we have only confirmed the regulation of VER on the PI3K/AKT signaling pathway, while its deeper regulatory mechanisms require further investigation. Additionally, we did not explore the effects of VER on other signaling pathways, such as MAPK, Wnt/β-catenin, and NF-κB, which limits our comprehensive understanding of its mechanisms of action. Therefore, future research will require additional experiments to validate the potential impacts of VER on these related pathways.

To sum up, our findings indicate that VER inhibits the growth, migration, and invasion of OS cells, enhances cell death, and demonstrates significant anti-OS activity *in vivo*. This suppressive effect could be linked to the PI3K/AKT/mTOR signaling pathway. More research is needed to understand the anti-tumor properties of VER and to progress the development of therapeutic methods for OS.

In conclusion, VER can reduce the growth, movement, and spread of OS cells and trigger cell death both *in vitro* and *in vivo* by blocking the PI3K/AKT pathway. Furthermore, VER has little toxicity and adverse effects *in vivo*, making it a promising candidate for OS treatment.

## CRediT authorship contribution statement

**Zhou Xie:** Writing – original draft, Project administration, Investigation, Conceptualization. **Xiao Qu:** Writing – review & editing, Methodology, Data curation. **Ziyun Li:** Visualization, Investigation. **Yingtao Duan:** Methodology, Data curation. **Yafei Zhu:** Visualization, Investigation. **Jiayu Wang:** Methodology, Data curation. **Xueqian Han:** Methodology, Data curation. **Jun Zhang:** Supervision, Funding acquisition. **Jinyong Luo:** Supervision, Resources, Funding acquisition. **Xiaoji Luo:** Supervision, Funding acquisition, Data curation, Conceptualization.

## Data availability

The data will be made available on request.

## Funding

This research was supported by The First Affiliated Hospital of Chongqing Medical University’s “Discipline Summit Plan” (No. cyyy-xkdfjhcgzh-202303), Chongqing Young and Middle-aged Medical High-end Talent Studio (China) (No. cstc2022ycjh-bgzxm0103), China Postdoctoral Science Foundation (No. 2024MD754029), and Chongqing Natural Science Foundation Project (China) (No. CSTB2024NSCQ-MSX1218).

## Conflict of interests

The authors declare that they have no known competing financial interests or personal relationships that could have appeared to influence the work reported in this paper.
