# Supplementary material for: Veratramine influences the proliferation of human osteosarcoma cells through modulating the PI3K/AKT signaling cascade
Source: Genes Dis. 2025 Apr 11;13(1):101630. doi: 10.1016/j.gendis.2025.101630 (PMC12555783; doi:10.1016/j.gendis.2025.101630)
Supplement: Multimedia component 1 [file mmc1.docx]

**Supplementary Table1. Summary of Veratramine: Administration, Disease Type, and Adverse Effects in Animal Studies**

| Drug | Disease Type | Administration Route | Animal Species | Dosage and Frequency | Adverse Effects | Title of the Literature | Reference DOI |
| --- | --- | --- | --- | --- | --- | --- | --- |
| Veratramine | liver cancer | Tail vein injection | BALB/c nude mice | 2mg/Kg, three times a week for 4 weeks. | no obvious systemic toxicity | Veratramine suppresses human HepG2 liver cancer cell growth in vitro and in vivo by inducing autophagic cell death | DOI: 10.3892/or.2020.7622 |
| Veratramine | Androgen-Independent Prostate Cancer | Intraperitoneal injection | BALB/c nude mice | 1.5 and 3 mg/kg twice a week for 4 weeks. | without any toxicity | Veratramine Inhibits the Cell Cycle Progression, Migration, and Invasion via ATM/ATR Pathway in Androgen-Independent Prostate Cancer | DOI: 10.1142/S0192415X2350060X |
| Veratramine | liver cancer | Intragastric | BALB/c nude mice | 10 mg/kg, every day for 3 weeks | not reported | Pharmacogenomic profiling of intra-tumor heterogeneity using a large organoid biobank of liver cancer | DOI: 10.1016/j.ccell.2024.03.004 |
| Veratramine | Osteosarcoma | Intragastric | BALB/c nude mice | 20 30 40mg/Kg，QOD (Quaque Omnis Die) | no obvious systemic toxicity | in our study | / |

**Supplementary Table2. Affinity between Veratramine and core targets**

| Ligand | Receptor | PDB ID | Binding Affinity(kJ/mol)) |
| --- | --- | --- | --- |
| Veratramine | AKT1 | 4EJN | -10.8 |
|  | HSP90AA1 | 3QTF | -10.8 |
|  | BCL2 | 6V4M | -7.6 |
|  | EGFR | 6LUB | -8.9 |
|  | CCND1 | 2W96 | -7.6 |
|  | PIK3CA | 7JIU | -9 |
|  | JAK2 | 8C09 | -8.7 |
