# Supplementary material for: Veratramine influences the proliferation of human osteosarcoma cells through modulating the PI3K/AKT signaling cascade
Source: Genes Dis. 2025 Apr 11;13(1):101630. doi: 10.1016/j.gendis.2025.101630 (PMC12555783; doi:10.1016/j.gendis.2025.101630)
Supplement: Multimedia component 2 [file mmc2.pdf]

重庆医科大学动物实验伦理审查同意书

批准编号:IACUC-CQMU-2024-0576

项目名称:藜芦胺对人骨肉瘤细胞的影响及其机制研究的实验方案

项目来源：研究生自选课题

项目负责人:罗小辑

负责人单位:重庆医科大学附属第一医院

申请日期:2024年04月26日

批准日期:2024年07月18日

本项目所涉及的动物实验方案经过重庆医科大学实验动物管理和使用委员会（IACUC-CQMU）审查，符合动物保护、动物福利和伦理原则，符合国家实验动物福利伦理的相关规定，同意按此方案进行实验。

重庆医科大学实验动物管理和使用委员会

2024年07月18日

IACUC Approval Certification

Approval number:IACUC-CQMU-2024-0576

Project title:Experimental protocol for the study of the effect of veratramine on human osteosarcoma cells and its mechanisms

Funding source:/

Principal investigator:Xiaoji Luo

Department:The First Affiliated Hospital of Chongqing Medical University

Application date:2024-04-26

Approval date:2024-07-18

All animal procedures to be employed in the project was approved by Institutional Animal Care and Use of Chongqing Medical University (IACUC-CQMU).

Jun Yuan

Jun Yuan， MD
Chair, IACUC
Chongqing Medical University
